# Web3 and digital mental health: Opportunities to scale sustainable mental health promotion and peer support

**DOI:** 10.3389/fpsyt.2022.945830

**Published:** 2022-07-22

**Authors:** Johannes Thrul, Luther G. Kalb, Patrick H. Finan, Zachary Prager, John A. Naslund

**Affiliations:** ^1^Department of Mental Health, Johns Hopkins Bloomberg School of Public Health, Baltimore, MD, United States; ^2^Sidney Kimmel Comprehensive Cancer Center at Johns Hopkins University, Baltimore, MD, United States; ^3^Centre for Alcohol Policy Research, La Trobe University, Melbourne, VIC, Australia; ^4^Center for Autism & Related Disorders, Kennedy Krieger Institute, Baltimore, MD, United States; ^5^Department of Psychiatry & Behavioral Sciences, Johns Hopkins University School of Medicine, Baltimore, MD, United States; ^6^Mindful Labs, San Diego, CA, United States; ^7^Department of Global Health and Social Medicine, Harvard Medical School, Boston, MA, United States

**Keywords:** peer support, Web3, digital health, mental health, health promotion

The global COVID-19 pandemic has demonstrated the need for accessible, flexible, scalable, and sustainable mental health treatment options. One of the clear success stories of the pandemic was how quickly healthcare systems and governments removed regulations to allow a rapid transition to remote delivery of mental health services. This nimbleness, however, exposed the growing scarcity of the mental health provider workforce. For instance, 61% of practicing psychiatrists in the US are nearing retirement (over 55 years old) (1), and the demand for college counselors has quickly outpaced the availability of university mental health services (2). The Department of Health and Human Services projected a protracted national workforce shortage in all mental health professionals by 2025 (3). Critically, those estimates did not take into account the mental health toll of the pandemic. Beyond provider shortage, other long-standing barriers to mental healthcare include costs of care, with the latter being exacerbated by the large number of providers who only accept out-of-pocket payments (4), as well as inequitable distribution of mental health providers with especially limited access to effective and quality care among racial and ethnic minority groups (5, 6). Remote delivery of mental health services may help to address some barriers to accessing care based on where patients live; yet, it will likely not fully solve the significant issues of provider shortage, rising costs, and lack of culturally appropriate services. New models of mental health delivery and funding are urgently needed.

## Peer support

Supported by both the Substance Abuse and Mental Health Services Administration and the Centers for Disease Control (7, 8), peer support has emerged as an evidenced-based approach to promote recovery from both mental and substance use disorders (9). Individuals with lived experience are critical to this model, providing peer support based on their expertise in managing their own conditions, while also receiving community support in return. Core principles of peer workers involve delivering recovery-oriented, person-centered, trauma-informed care (7). Peer support is particularly valuable for reaching underserved, marginalized groups like LGBTQ+ youth, as well as reduction in service use disparities among racial and ethnic minority youth (10). Fortunately, research has shown peer support programs are effective when delivered remotely and in a digital format (9).

However, despite its promise, peer support faces sustainability and scalability challenges that may limit its impact. Peer support workers can often be required to volunteer their time in the provision of services, or receive minimal compensation, as many services are not yet widely reimbursable, despite new legislation solidifying the role of peer support workers at the national level. Furthermore, to effectively train and supervise a peer support workforce, which is essential for achieving the scalability, dedicated resources are needed to ensure appropriate infrastructure is in place. Digital approaches hold potential for overcoming many of these challenges, yet barriers persist in ensuring access to quality, affordable, and effective services. To optimize peer support in the digital space, a model that prioritizes meaningful remuneration for meaningful peer support contributions may be necessary.

## Web3

Over the last several decades, the field of computer science has ushered in a new financial paradigm through blockchain-based technologies. With a near 3 trillion dollar market capitalization in November 2021, and a White House Executive Order that opens the doors to regulation, blockchain-based technologies—including cryptocurrencies and a wide range of other crypto-assets—are now becoming mainstream (11). By building on the open and immutable digital ledger technology of the blockchain, Web3 presents a fundamentally new way to design and structure mental healthcare delivery systems.

The concept of Web3 has emerged as an evolution from Web 1.0 and Web 2.0. Web 1.0 represented the nascent state of the internet, where websites were static, read-only entities that were owned by companies with little user interaction or input. This offered informational resources for patients with mental health questions and concerns, but lacked an interactive component necessary for mental healthcare delivery. Our internet today, or Web 2.0, is a more dynamic (read-write) and open-source (user created content) environment. This allows for interaction between patients and providers and has fostered the development of a variety of mobile mental healthcare delivery options.

The prevailing business models of Web 2.0 are fundamentally advertisement-based. They are designed to generate revenue off of user data and attention based on behavioral monitoring algorithms (12). This approach is well-aligned for corporate profit generation, but poorly aligned for the incentivization of users. This makes it a poor fit to sustain a peer support worker economy. Further, the algorithmic design of Web 2.0 plays a central part in increasing mistrust and misinformation, political polarization (13), and worsening mental health (14).

Web3 presents a new model, as read-write-own (15). It envisions an internet that is decentralized (ownership distributed among creators and users), permissionless (equal access to content), and trustless (via open-source, consensus-driven code that does not rely on a centralized or third party). Interaction in Web3 is supported by native, digital, cryptographically-defined assets that drive economic activity, including both rewards and payments. Just as important as economics, Web3 offers community building and empowerment, counteracting loneliness and societal dissolution.

## Bringing together digital peer support and Web3

Web3 and peer support are a natural marriage. Like peer support, Web3 is a member (rather than industry or government)-driven phenomenon. Because individual members share ownership in this community and its assets, Web3 may encourage and promote community-minded behaviors and mutually supportive interactions. It also offers an opportunity to deepen and sustain communities whose mutual goals are socially beneficial, such as promoting positive mental health. This can be seen in the developmental of Decentralized Autonomous Organizations (DAOs), which are member-owned and often governed by consensus-based decision making (16). The rules and policies governing DAOs are written in transparent (open source) smart contracts that cannot be changed by a single individual (decentralized), and are autonomously executed on the blockchain. In essence, DAOs democratize decisions that affect the network. Two recent examples of DAOs include the ConstitutionDAO (17), which raised over $40 million dollars in less than a week in a narrowly lost bid to purchase an original copy of the US constitution, and the Ukrainian DAO, which has donated nearly $7 million dollars to Ukraine (18).

A prevailing model of Web3 applications provides a funding mechanism for users to exchange content created within the community for a community-approved crypto-asset that holds real economic value and can be traded (for US dollars) on exchanges. This is critical since this novel currency can be spawned for activity and governed by the community (via DAOs). It is not reliant on transient federal, state, or private funds. Nor does it require lengthy, contentious legislation to allocate such funds. Three parties stand to financially gain from this model: (1) authors of the network; (2) peer workers who spend time helping users; and (3) users themselves, who could be rewarded for meeting treatment or health behavior change goals. Code of conduct of peer support workers could be observed and enforced through practice guidelines, community moderation, as well as supervisors/moderator that would also receive incentives for their contributions.

The incentive structure offered by Web3 could be transformative for mental healthcare. It rewards innovation, promotes the best peer support workers (through network effects, which can diminish bad actors/support overtime), and adherence to positive behavior change goals. It also has the potential to reduce economic disparities, which is a primary risk factor for mental distress during the COVID-19 pandemic (19), through financial rewards. If properly executed, the rewards tip the scale toward the network. This means peer supporters and users could stand to economically gain from expansion of the network. This is a vastly different approach than relying on corporations to identify how to profit from delivering mental health care or governments agreeing on allocation of tight budgets. In fact, it may even provide particular value to rural and international regions with little resources.

## Preparing for pitfalls and looking forward

Web3 holds substantial promise, but there are both challenges and hazards ahead. First, there must be a meaningful interaction between computer science, mental health experts, and individuals with lived experience. Programmers are needed to develop the infrastructure, while mental health practitioners must promote evidenced-based interventions and monitor the network for ethical implementation. Engagement of individuals with lived experience is also essential early on in establishing the Web3-mental health space in order to avoid replicating the same top-down and exclusionary practices that have created immense challenges to effectively reaching patients and delivering mental health services within existing health care systems. These parties must work closely to navigate a quickly changing technological, legal, and ethical landscape. Second, Web3 and blockchain technology do not eliminate structural barriers to inclusion, gender, and racial equity, and there remains a need to prioritize an inclusive framework to mental health research and healthcare. This could be realized by establishing clear standards and guidelines for delivery of services in the Web3-mental health space, as well as rewarding providers for offering more inclusive services, and incentivizing efforts to offer supervision and constructive feedback to peer support workers to promote ongoing skill development and quality assurance, while instilling greater confidence among end users. In an ideal Web3 mental health space, peer support workers will have prospects for personal growth and career progression, which could be incentivized through length of service, quality of care delivery, number of users supported, and gaining additional skills through training or education opportunities made available through Web3. Third, crypto-assets have repeatedly demonstrated high volatility, which could impact the attractiveness of incentives for peer support workers. This can be seen in the recent downturn, as of Summer 2022, where the overall cryptocurrency market capitalization has dropped by 2 trillion dollars. Fourth, crypto-assets require use of digital wallets to send and receive funds. Although the wallets are generally free, there are substantial security risks that have yet to be resolved, and there is a high barrier to entry, particularly for those without the necessary means or digital literacy skills to access and navigate such technologies.

It is also important to highlight the significant ethical issues in the cryptocurrency space that must be addressed prior to intersecting Web3 and mental health. In just the beginning of 2022, there have been instances of hacked block chain networks (20), locking of cryptocurrency assets within a centralized lending platform (21), use of extreme leverage in trading which increases crypto volatility (22), and the collapse of a very large (by market capitalization) cryptocurrency, including its algorithmic stable coin (23). This has created an environment that is quite uncertain without clearer US regulatory involvement.

Safeguarding the network and protecting users is a must, especially for first movers in this Web3-mental health space. This means that pioneers must be very careful in selecting their blockchain networks. Leveraging those with an established history may be preferred. Use of DAOs and specialized security companies can also afford some protections. It is also important that users of the network be very clear on the promises and drawbacks of using cryptocurrencies to power the Web3-mental health innovation. One way to overcome significant losses is that networks only use tokens as rewards and airdrops, limiting purchases to investors only. Moreover, a blockchain network using energy-efficient Proof-of-Stake (PoS) consensus mechanisms would be preferable over Proof-of-Work (PoW) to minimize the environmental impact (24). Lastly, the mantra of technologists often involves the idea to “move fast and break things.” Following such an approach could be disastrous, as it will put human lives at risk. Having a team of ethicists, clinicians, public, and mental health experts, individuals with lived experience, as well as computers scientists will be needed for successful implementation.

Web3 and healthcare are already intersecting. Blockchains and cryptocurrencies are being examined as a way to improve HIV outcomes in Kenya (25), fund cancer research (26), promote healthy behaviors through tracking of health data via wearable devices (27), and secure digital medical records (28). It is time we look to leverage this promise to improve global mental health.

## Author contributions

JT and LGK: initial draft and review and editing. PHF, ZP, and JAN: review and editing. All authors contributed to the article and approved the submitted version.

## Conflict of interest

The authors declare that the research was conducted in the absence of any commercial or financial relationships that could be construed as a potential conflict of interest.

## Publisher's note

All claims expressed in this article are solely those of the authors and do not necessarily represent those of their affiliated organizations, or those of the publisher, the editors and the reviewers. Any product that may be evaluated in this article, or claim that may be made by its manufacturer, is not guaranteed or endorsed by the publisher.
